# Application of the Theory of Planned Behavior to Predict Dental Attendance and Caries Experience among Children of Newcomers

**DOI:** 10.3390/ijerph16193661

**Published:** 2019-09-29

**Authors:** Maryam Amin, Maryam Elyasi, Babak Bohlouli, Mohamed ElSalhy

**Affiliations:** 1 School of Dentistry, Faculty of Medicine and Dentistry, University of Alberta, Edmonton, AB T6G 2R7, Canada; elyasi@ualberta.ca; 2 Department of Emergency Medicine, Faculty of Medicine and Dentistry, University of Alberta, Edmonton, AB T6G 2R7, Canada; bohlouli@ualberta.ca; 3 College of Dental Medicine, University of New England, Portland, ME 04103, USA; melsalhy@une.edu

**Keywords:** dental attendance, children, structural equation modeling, theory of planned behavior

## Abstract

*Background:* This study aimed to explore the predictors of regular dental attendance behavior and caries experience among children of newcomers to Canada using the Theory of Planned Behavior (TPB). *Methods:* A total of 274 newcomer parents with a child aged 1 to 12 years old who had lived in Canada for 10 years or less participated in this cross-sectional study. Children underwent a dental examination to measure their deft/DEFT, and parents completed a self-administered questionnaire. Parental attitudes, subjective norms, perceived behavioral control (PBC), and intention were examined using Structural Equation Modeling (SEM) as predictors of dental attendance behavior and caries experience using the TPB model. *Results:* Out of all the components of the model, attitude and PBC significantly predicted the intention (*p* < 0.05) while the subjective norm had no statistical effects on the loading of the model (*p* > 0.05). Intention significantly predicted behavior, but behavior could not predict caries experience. Although 51.6% of the variance of intention was predicted by this model, only 0.2% of the variance for dental attendance behavior was explained. *Conclusions*: The theory of planned behavior was successful in predicting dental attendance intention and behavior for children among newcomers, but this behavior could not predict caries experience in children.

## 1. Introduction

Newcomers form a sub-segment of the community that requires special attention due to their unique challenges, like financial insecurity, unemployment, language barriers, and cultural diversity [1,2]. Such differences can also predispose them to suboptimal oral health and oral health-related habits, including care-seeking behavior [3]. Regular dental attendance is a preventive measure to optimize children’s oral health [4]. Such habits must be established early after migration to prevent oral health deterioration until families are more settled. However, lower rates of dental visits have been repeatedly reported among newcomers [1,5]. Patient-level factors affecting children’s dental attendance include parents’ education, socioeconomic status, behavioral beliefs, perceived power, and subjective norms [6].

Health behaviors can be predicted and explained by psychosocial theories. The theory of planned behavior (TPB) is one of the applied theories used to predict determinants of health behaviors and develop the interventions used in health promotion programs and behavioral changes [7]. TPB is a socio-cognitive model of the attitude–behavior relationship mediated by behavioral intentions. According to TPB, intention directly influences behavior and is shaped by attitudes, subjective norms, and perceived behavioral control of behavior [8]. TBP has been successful in explaining dental attendance behavior via attitude and intention in the general population [9,10]. However, little is known about the usefulness of this theory in high-risk groups, such as newcomers to the country, where the relationship between attitude and intention or intention and behaviors may not be as straightforward as in the proposed theory.

The cultural beliefs and perceptions that immigrants bring with them, as well as the level of their acculturation and the way they integrate into their new surroundings, may also influence their care-seeking behavior [11]. Newcomers are transitioning from their original culture into a new culture whose social norms may contradict their traditional cultural norms, and their perceived behavioral control may be influenced by challenges like financial insecurity, unemployment, and language barriers [11,12]. This study aimed to explore the predictors of regular dental attendance behavior and caries experience among children of newcomers using the Theory of Planned Behavior as a theoretical framework.

## 2. Methods

### 2.1. Setting and Sampling

A convenience sample group of immigrant parents with a child age 1–12 who had lived in Canada for 10 years or less was invited to participate in this cross-sectional study. A group of newcomer families and their children were recruited through programs run by the community settlement agencies serving newcomers in Edmonton. All participants signed an informed consent form before participation. The study protocol was approved by the University of Alberta Research Ethics Board and Alberta Health Services (Protocol # Pro00058397).

### 2.2. Data Collection

Data collection was done in different community locations through events organized by community settlement agencies. Trained bilingual community workers obtained parents’ consent and helped with the administration of the questionnaire, which was in English. Almost all parents who were approached accepted the invitation. The questionnaire, developed based on previous studies [13,14], was first tested in a small focus group of 20 mothers with young children to determine the comprehensiveness, understandability, and neutrality of the questions, and required modifications were made. The questionnaire was composed of 2 major sections. The first section included participants’ demographics and children’s oral hygiene and dietary habits. Collected demographics included the child’s and mother’s age, the number of children in the family, the child’s live-in parent status, the child’s place of birth, number of years in Canada, the mother’s level of education, household monthly income, and having dental insurance. Questions on the children’s oral hygiene and diet included the frequency of brushing (less than twice a day/twice a day or more), the last dental visit (within the past 12 months/over one year/never), the reason for their last dental visit (regular check-up/urgent dental problems/others), and sugar consumption frequency (never/less often than everyday/once a day/twice a day/three times a day or more).

The second section was a validated questionnaire consisting of 24 items based on the Theory of Planned Behaviour (TPB) constructs adopted to examine parental attitudes (8 items), subjective norms (10 items), perceived behavioral control (PBC) (5 items), and intention (1 item) towards their child’s dental attendance. Attitude questions included statements about dental visits being traumatic, important, reassuring, unpleasant, and/or if a dental visit decreases caries experience and/or reduces fear toward the dentist. Subjective norms were the evaluated norms based on visiting the dentist early among partners, parents, other family members, friends, and/or family physicians. The perceived control section examined having time, positively preparing the child, and the ability to manage a dental visit twice a year. The intention was measured by the statement “I think of making an appointment with the dentist on time.” Responses for the questions were measured on a 7-point Likert scale ranging from 1 (strongly disagree) to 7 (strongly agree). The sum of the responses to the items measuring the TPB constructs indicates their final scores. Therefore, a higher total score for items measuring a construct indicated a more positive/stronger construct.

Completion of the questionnaire took about 20 to 30 min. Questionnaires were assessed for completeness on-site, and any missing or incomplete information was immediately collected from the participants.

### 2.3. Dental Examination

A portable dental chair, LED lamp, sterilized mirror, and explorer were used for dental examination of children in the community. The caries status of each child was recorded according to WHO criteria [15] by a calibrated dentist. Caries experience was measured as the total number of decayed teeth, teeth extracted due to caries, and filled teeth (deft/DEFT).

### 2.4. Statistical Analysis

Data analyses were performed using the SPSS 24.0 software (IBM Corp., Armonk, NY, USA). Means, standard deviations, and Pearson’s correlations were computed between the study variables. Discrete variables were reported in percentages, and continuous variables were presented as the mean ± SD, the median, and the range, and compared with the *t*-test (where appropriate). Cronbach’s alpha values were calculated to report an estimate of the internal consistency among items of TPB constructs, including attitude, subjective norm (SN), and perceived behavior control (PBC). A confirmatory factor analysis was performed to examine the prior model’s (TPB) goodness of fit with the study cohort. A *p*-value of less than 0.05 was considered significant in the analyses.

SPSS AMOS 7.0 (IBM Corp., Armonk, NY, USA) was used for Structural Equation Modeling (SEM) to examine the predictive ability of TPB constructs to predict dental attendance behavior and, subsequently, caries experience. The maximum likelihood was used to estimate the parameters of the model [16]. The adequacy of the model fit was examined using the chi-square test statistic, the comparative fit index (CFI), and the root mean squared error of approximation (RMSEA) [16,17].

## 3. Results

### 3.1. Participates Characteristics

In this study, 274 parents completed the questionnaires with no missing data in the final dataset. 25.6% of the families were from South Asia (Nepal, India, Burma, Moldova), 23% were from East Asia (China, Hong Kong, Philippines), 38.2% were from Africa (Eritrea, Ethiopia, Somalia), and 11.8% were from East Europe (Ukraine, Russia, Romania). The mean (SD) age of the children was 4.5 years (SD = 2.9), and 55% were girls. There was no significant age difference according to gender (*p* > 0.05). About 55% of children were born in Canada, and 85.7% of them were living with both parents. The mean age of the mothers was 34.9 years (SD = 6.3), and 57.2% had college or higher education. Of 274 families, 41% had lived in Canada for less than 5 years and 37.5% reported a monthly household income of less than $2000. The majority of the participants were from China, India, Nepal, Ethiopia, and Eritrea. Of the 274 children, 42.3% had dental insurance, 34.3% visited the dentist for check-ups within a year, 62.8% brushed their children’s teeth less than once a day, and 34.7% consumed sugar twice a day or more. The mean deft/DEFT was 3.3, ranging from 1 to 16. The participants’ characteristics are summarized in Table 1.

### 3.2. Scale Reliability

A subset of items designed to measure TBP constructs was assessed as follows: attitude through eight 7-point Likert-type items (Cronbach’s: 057); subjective norm through nine 7-point Likert-type items (Cronbach’s: 0.85); and perceived behavioral control through five 7-point Likert-type items (Cronbach’s: 0.62). Given the preliminary reliability values, some items were removed to improve the Cronbach alpha value; therefore, by removing 2 items from the PBC and 2 items from the attitude constructs, the Cronbach alpha increased to 0.738 and 0.742 respectively. All items measuring a subjective norm construct were kept in the analyses. Internal consistency coefficients of more than 0.70 are generally considered acceptable [18]. Taking into account the sensitivity of Cronbach’s alpha to a low number of items, the average Cronbach’s alpha was over 0.88 and suggested that the scales are homogenous (Appendix A).

### 3.3. Structural Equation Modeling

In the first step, confirmatory factor analyses showed a significant covariance between the latent variables suggesting that the TPB model was appropriate for the study population to predict children’s dental attendance behavior and caries experience (Table 2).

**Figure 1:**
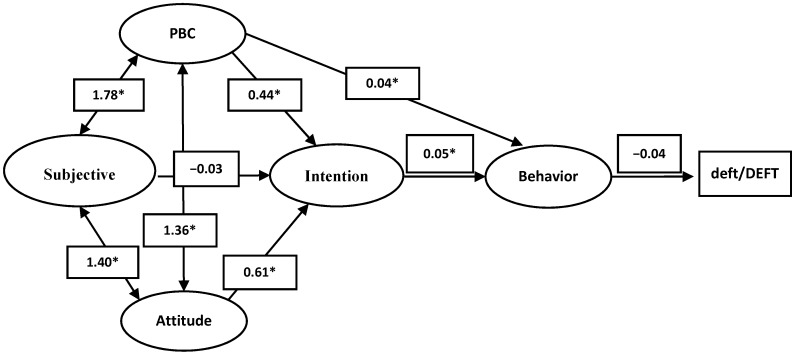
Causal model path beta coefficients, * *p* < 0.05.

The SEM showed an acceptable fit based on the measured indices (c² = 376.970, df = 177, *p* < 0.001, CMIN/DF = 2.13, RMSEA = 0.06, CFI = 0.9). The SEM results showed that, out of all components of the model, the PBC and attitude significantly predicted the intention (*p* < 0.05), and the subjective norm had no statistical effects on the loading of the model. Intention also significantly predicted behavior (*p* < 0.05), but behavior could not predict deft/DEFT. Additionally, there was a significant association between the PBC and behavior in the model (Table 3). A diagram of measurements and structural components of the model is illustrated in Figure 1. Standardized beta coefficients for the measurement and structural parts of the model components are presented in Table 3. The actual diagram of the SEM analyses is presented in Appendix B. In this model, while 51.6% of the variance accounted for intention, only 0.2% and 0.1% of the variances explained dental attendance behavior and deft/DEFT, respectively.

## 4. Discussion

This study applied TPB as a model to predict regular dental attendance behavior and caries experience among newcomers’ children using a Structural Equation Modeling approach. The attitude and PBC components of TPB significantly predicted parental intention to have regular dental visits for their children. Subjective norms had no statistical effects on the loading of the model. Intention also significantly predicted dental attendance behavior, but the behavior could not predict caries experience. Only 0.2% of the variance in dental attendance behavior was explained by intention.

The proportion of dental attendance within the previous year was about 34% among children of newcomers. This prevalence is lower than the previously reported 51% among 378 preschoolers in Edmonton, Alberta [13]. A higher proportion of participants in the present study reported a monthly income of less than 1999 CAD compared to the general population (37.5% vs. 21.6%). In addition, participants in the present study had lower rates of dental insurance compared to the general population (42.3% vs. 74.8%). This could be an explanation for the differences in dental attendance rates. Dental costs and insurance were the most commonly reported reasons behind suboptimal use of dental services, as they can restrict access to dental care [6].

Attitudes toward different behaviors have always been considered one of the strongest determinants of these behaviors [7]. In the present study, both attitude and PBC accurately predicted intention. This finding supports other studies that predict decisions to utilize dental care [9,10]. This highlights the importance of oral health education and promotion programs targeting both attitude and PBC toward dental attendance [19]. Interestingly, the subjective norm construct did not predict intention. A potential explanation is that newcomers are transitioning from their original cultures into a new culture, where subjective norms may contradict new cultural norms [11].

Intention was translated into a behavior among participants of the present study. Although the pathway was statistically significant, the variance of behavior explained in the model was very low (0.2%). This could be attributed to the minimal number of items adopted in the study to measure participants’ intentions in the present study. In addition, the presence of socioeconomic and/or structural barriers may affect the translation of intention to behavior [7]. Financial insecurity, unemployment, language barrier, transportation, and navigating the healthcare system have been reported as factors affecting care-seeking behavior among newcomers [6]. There is no conclusive literature on which specific barriers will mostly affect the translation of intention to behavior [7]. However, this difference highlights the importance of affordable, culturally appropriate programs that focus on vulnerable populations to prevent deterioration of their oral health and keep them aligned with the majority population.

Regular dental attendance provides an opportunity to assess patients’ caries risk, provide oral health education, and provide professional preventive care and treatments. Having a dental visit is a proxy measure of access to dental care. Although a routine biannual dental visit is the most recommended practice, this practice has been challenged because of the lack of evidence [4]. Multiple Cochrane reviews concluded that there is the lack of evidence to support or refute the benefits of dental check-ups every 6 months or to demonstrate any harmful effects of longer recall intervals [19,20]. In the present study, regular dental attendance could not predict caries experience measured as deft/ DEFT. The complexity of caries etiology may be the main explanation for this result [21]. Diet, oral hygiene behaviors and patient susceptibility are important contributors to caries formation [21]. Although there is a lack of evidence regarding the benefits of specific recall intervals, accessing dental care might have a generally positive impact on quality of life [22]. It was shown that regular users of dental services have better OHRQoL compared to non-regular users [22].

While the TPB showed success in explaining dental attendance behavior through attitude and intention in the general population [9,10], its prediction was not strong among the immigrant population in this study (i.e., only 0.2% of the variance of dental attendance behavior was explained). Using a different health behavior model/theory and/or expansion of the TPB can be valuable to understand the determinants/predictors of dental attendance among newcomers. Some other behavioral constructs have been proposed to predict oral health behaviors and can be added to the model. The Sense of Coherence, for example, was a significant determinant of oral health-related behaviors, including tooth brushing frequency, daily smoking, and dental attendance [13,23]. Sense of Coherence (SOC) describes the capability of individuals to use existing resources in order to overcome difficulties and cope with life stressors to perform healthy behaviors [24]. Immigrant individuals with a stronger SOC may be able to transfer their intentions to behaviors. If this is true, newcomers may benefit from programs that improve SOC through skill training. However, this must be examined in future studies.

One limitation of the present study is its convenient sample group. Although all clients who attended the settlement agency activities were invited, the ones who participated might have been the most affected by dental care barriers. However, a random sampling of the immigrant population is not achievable as this population is scattered among the general population and across the province. Another limitation was the use of self-administered English language questionnaire that could affect participants’ understanding of the questions. To avoid misinterpretation of the questions, bilingual translators were available on-site to help the participants if needed.

SEM was used in the present study to examine how TPB constructs predict dental attendance behavior and caries experience. SEM has advantages over a basic multivariate regression analysis in its ability to simultaneously test the hypothesized relationships among observed and latent variables in the model. However, similar to other statistical methods, SEM has its limitations. Although a latent variable can be a closer approximation of a construct than a measured variable in the model, it may not be a clear representation of the construct [25]. Although we used the term effect while explaining the SEM results, this does not mean that our proposed Structural Equation Model is a causal model due to the cross-sectional design of this study. Future longitudinal studies will be needed to explore the causality effects of the TPB construct on the exhibition of the actual behavior instead of self-reported past behavior.

In conclusion, the theory of the planned behavior was successful in predicting regular dental attendance behavior among the children of newcomers, but their behavior could not predict caries experience. Parental attitude and PBC were the main predictors for intention to engage in regular dental attendance. Future studies should work on either expanding the TPB or use of theories/models that include interpersonal, community, or environmental constructs to better predict dental attendance behavior and caries experience among newcomers.

## Figures and Tables

**Table 1:** Participants characteristics (number of participants: 274).

Child’s age		deft/DEFT (Caries experience)	
Mean	4.53	Mean	3.28
Median	4	Median	2
SD (range)	2.98 (1–12)	SD (range)	3.76 (0–16)
**Children of a female gender** (N) (%)	150 (55.0)	**Children with dental insurance** (N) (%)	116 (42.3)
**Children born in Canada** (N) (%)	148 (54.9)	**Children’s last dental visit within a year** (N) (%)	94 (34.3)
**Mother’s age**		**Reason for visit** (N) (%)	
Mean	34.92	Check up	63 (23.0)
Median	34	Treatment	32 (11.6)
SD	6.35	**Tooth brushing frequency** (N) (%)	
		<twice/day	172 (62.8)
		≥twice/day	102 (37.2)
**Number of Children in family** (N) (%)		**Sugar intake frequency** (N) (%)	
≤2	218 (79.5)	≤once/day	179 (65.3)
3 or more	56 (20.5)	≥twice/day	95 (34.7)
**Mother’s education level** (N) (%)		**Monthly income** (N) (%)	
High school	117 (42.7)	≤1999	103 (37.5)
College	51 (18.6)	2000–3999	107 (39.0)
University	106 (38.6)	≥4000	64 (23.3)
**Living with both parents**		**Years in Canada** (N) (%)	
N (%)	235 (85.7)	≤5 years	112 (40.9)

**Table 2:** Covariance between model components.

			Estimate	S.E.	C.R.	P
PBC	<-->	Subjective Norm	1.788	0.246	7.274	0.001
PBC	<-->	Attitude	1.366	0.188	7.247	0.001
Subjective Norm	<-->	Attitude	1.405	0.174	5.630	0.001

**Table 3:** Standardized regression weight for the structural parts of the Theory of Planned Behavior (TPB) model components.

Items			Estimate	SE	CR	*p*-Value
PBC_1	<--->	PBC	0.579	0.099	8.861	0.001
PBC_2	<--->	PBC	0.757			
PBC_3	<--->	PBC	0.774	0.076	11.698	0.001
Subjective norm_1	<--->	Subjective norm	0.604	0.094	8.676	0.001
Subjective norm_2	<--->	Subjective norm	0.731	0.099	10.179	0.001
Subjective norm_3	<--->	Subjective norm	0.658			
Subjective norm_4	<--->	Subjective norm	0.635	0.071	12.074	0.001
Subjective norm_5	<--->	Subjective norm	0.704	0.103	9.877	0.001
Subjective norm_6	<--->	Subjective norm	0.166	0.087	3.107	0.002
Subjective norm_7	<--->	Subjective norm	0.472	0.098	6.965	0.001
Subjective norm_8	<--->	Subjective norm	0.399	0.094	6.371	0.001
Subjective norm_9	<--->	Subjective norm	0.436	0.11	6.466	0.001
Attitude_1	<--->	Attitude	0.609	0.091	10.127	0.001
Attitude_2	<--->	Attitude	0.601	0.089	9.969	0.001
Attitude_3	<--->	Attitude	0.842			
Attitude_4	<--->	Attitude	0.29	0.101	4.482	0.001
Attitude_5	<--->	Attitude	0.593	0.077	10.407	0.001
Last child dental visit	<--->	Behavior	−0.228	0.292	−0.208	0.835
Reason for dental visit	<--->	Behavior	3.321			
Intention	<--->	Subjective norm	−0.301	0.241	−1.598	0.11
Intention	<--->	Attitude	0.61	0.167	5.032	0.001
Intention	<--->	PBC	0.441	0.174	2.904	0.004
Behavior	<--->	PBC	0.042	0.022	1.993	0.046
Behavior	<--->	Intention	0.049	0.019	−2.365	0.018
DEFT/deft	<--->	Behavior	−0.037	0.439	−0.197	0.844

## Appendix A. Scale Reliability Measure

**Item**	**Scale Mean If Item Deleted**	**Scale Variance If Item Deleted**	**Corrected Item-Total Correlation**	**Squared Multiple Correlation**	**Cronbach’s Alpha If Item Deleted**
PBC_1	85.01	360.271	0.436	0.380	0.884
PBC_2	84.31	356.023	0.576	0.498	0.878
PBC_3	83.91	357.743	0.653	0.585	0.876
Subjective norm_1	83.99	362.047	0.548	0.446	0.879
Subjective norm_2	84.08	353.942	0.656	0.513	0.876
Subjective norm_3	84.70	349.850	0.638	0.577	0.876
Subjective norm_4	84.85	357.151	0.620	0.547	0.877
Subjective norm_5	84.25	351.559	0.653	0.464	0.875
Subjective norm_6	85.15	358.736	0.478	0.593	0.882
Subjective norm_7	84.92	367.513	0.427	0.309	0.884
Subjective norm_8	84.45	353.897	0.591	0.524	0.878
Subjective norm_9	84.83	359.649	0.466	0.540	0.883
Attitude_1	83.91	363.959	0.494	0.374	0.881
Attitude_2	84.32	367.492	0.460	0.392	0.883
Attitude_3	83.49	364.573	0.644	0.554	0.877
Attitude_4	84.78	384.936	0.195	0.242	0.892
Attitude_5	83.91	364.703	0.554	0.486	0.879

## References

[1] Amin M., Perez A., Nyachhyon P. (2013). Parental Awareness and Dental Attendance of Children Among African Immigrants. J. Immigr. Minority Health Cent. Minor. Public Health.

[2] Vargas C.M., Ronzio C.R. (2006). Disparities in early childhood caries. BMC Oral Health.

[3] Gao X.L., McGrath C. (2011). A review on the oral health impacts of acculturation. J. Immigr. Minor. Health Cent. Minor. Public Health.

[4] Sheiham A. (1977). Is there a scientific basis for six-monthly dental examinations?. Lancet.

[5] Newbold K.B., Patel A. (2006). Use of dental services by immigrant Canadians. J. Can. Dent. Assoc..

[6] Badri P., Saltaji H., Flores-Mir C., Amin M. (2014). Factors affecting children’s adherence to regular dental attendance: A systematic review. J. Am. Dent. Assoc..

[7] Armitage C.J., Conner M. (2001). Efficacy of the Theory of Planned Behaviour: A meta-analytic review. Br. J. Soc. Psychol..

[8] Ajzen I. (1991). The theory of planned behavior. Organ. Behav. Hum. Decis. Process..

[9] Astrom A.N., Lie S.A., Gulcan F. (2018). Applying the theory of planned behavior to self-report dental attendance in Norwegian adults through structural equation modelling approach. BMC Oral Health.

[10] Luzzi L., Spencer A.J. (2008). Factors influencing the use of public dental services: An application of the Theory of Planned Behaviour. BMC Health Serv. Res..

[11] Dahlan R., Badri P., Saltaji H., Amin M. (2019). Impact of acculturation on oral health among immigrants and ethnic minorities: A systematic review. PLoS ONE.

[12] Amin M., ElSalhy M. (2017). Factors Affecting Dental Attendance of Children of New Immigrant Parents: A Cross-Sectional Study. J. Immigr. Minority Health Cent. Minor. Public Health.

[13] Elyasi M., Abreu L.G., Olsen C., Baker S.R., Lai H., Major P.W., Amin M. (2018). Parent’s Sense of Coherence and Children’s Oral Health-Related Behaviors: Is There an Association?. Pediatr. Dent..

[14] Van den Branden S., Van den Broucke S., Leroy R., Declerck D., Hoppenbrouwers K. (2013). Measuring determinants of oral health behaviour in parents of preschool children. Community Dent. Health.

[15] World Health Organization (2013). Oral Health Surveys: Basic Methods.

[16] Brown T.A. (2015). Confirmatory Factor Analysis for Applied Research.

[17] Hu L., Bentler P.M. (1999). Cutoff criteria for fit indexes in covariance structure analysis: Conventional criteria versus new alternatives. Struct. Equ. Model. A Multidiscip. J..

[18] Santos J.R.A. (1999). Cronbach’s alpha: A tool for assessing the reliability of scales. J. Ext..

[19] Beirne P., Clarkson J.E., Worthington H.V. (2007). Recall intervals for oral health in primary care patients. Cochrane Database Syst. Rev..

[20] Riley P., Worthington H.V., Clarkson J.E., Beirne P.V. (2013). Recall intervals for oral health in primary care patients. Cochrane Database Syst Rev..

[21] Fisher-Owens S.A., Gansky S.A., Platt L.J., Weintraub J.A., Soobader M.J., Bramlett M.D., Newacheck P.W. (2007). Influences on children’s oral health: A conceptual model. Pediatrics.

[22] Crocombe L.A., Broadbent J.M., Thomson W.M., Brennan D.S., Poulton R. (2012). Impact of dental visiting trajectory patterns on clinical oral health and oral health-related quality of life. J. Public Health Dent..

[23] Elyasi M., Abreu L.G., Badri P., Saltaji H., Flores-Mir C., Amin M. (2015). Impact of Sense of Coherence on Oral Health Behaviors: A Systematic Review. PLoS ONE.

[24] Antonovsky A. (1993). The structure and properties of the sense of coherence scale. Soc. Sci Med..

[25] Beran T.N., Violato C. (2010). Structural equation modeling in medical research: A primer. BMC Res. Notes.

